# Ten Previously Unassigned Human Cosavirus Genotypes Detected in Feces of Children with Non-Polio Acute Flaccid Paralysis in Nigeria in 2020

**DOI:** 10.3390/v17060844

**Published:** 2025-06-12

**Authors:** Toluwani Goodnews Ajileye, Toluwanimi Emmanuel Akinleye, Temitope O. C. Faleye, Lander De Coninck, Uwem Etop George, Anyebe Bernard Onoja, Sheriff Tunde Agbaje, Ijeoma Maryjoy Ifeorah, Oluseyi Adebowale Olayinka, Elijah Igbekele Oni, Arthur Obinna Oragwa, Bolutife Olubukola Popoola, Olaitan Titilola Olayinka, Oluwadamilola Gideon Osasona, Oluwadamilola Adefunke George, Philip G. Ajayi, Adedolapo A. Suleiman, Ahmed Iluoreh Muhammad, Isaac Komolafe, Adekunle Johnson Adeniji, Jelle Matthijnssens, Moses Olubusuyi Adewumi

**Affiliations:** 1 Department of Virology, College of Medicine, University of Ibadan, Ibadan 200212, Nigeria; ajileye017@yahoo.com (T.G.A.); bernardonoja@yahoo.com (A.B.O.); sheffydeen1@gmail.com (S.T.A.); debowaley2k@gmail.com (O.A.O.); oni.elijah002@gmail.com (E.I.O.); bolupopson@yahoo.com (B.O.P.); ajayiphilipg@gmail.com (P.G.A.); divinedola068@gmail.com (A.A.S.); adek1808@yahoo.com (A.J.A.); 2 Phytomedicine Unit, Department of Pharmacognosy, Faculty of Pharmacy, University of Ibadan, Ibadan 200005, Nigeria; toluwanimiemmanuel@rocketmail.com; 3 Center for Environmental Health Engineering, Biodesign Institute, Arizona State University, Tempe, AZ 85281, USA; 4 Laboratory of Viral Metagenomics, Department of Microbiology, Immunology and Transplantation, Rega Institute, Universiteit Leuven, 3000 Leuven, Belgium; 5 African Centre of Excellence for Genomics of Infectious Diseases, Redeemer’s University, Ede 232101, Nigeria; george27@run.edu.ng (U.E.G.); muhammadahmedilu@gmail.com (A.I.M.); 6 Department of Biological Sciences, Faculty of Natural Sciences, Redeemer’s University, Ede 232101, Nigeria; toshokomolafe@gmail.com; 7 Department of Medical Laboratory Science, Faculty of Health Science and Technology, College of Medicine, University of Nigeria Enugu Campus, Enugu 400241, Nigeria; ijeoma.ifeorah@unn.edu.ng; 8 Centre for Translation and Implementation Research, University of Nigeria, Nsukka 410001, Nigeria; 9 Department of Veterinary Microbiology, Faculty of Veterinary Medicine, University of Jos, Jos 930003, Nigeria; oragwaa@unijos.edu.ng; 10 National Polio Laboratory, College of Medicine, University of Ibadan, Ibadan 200212, Nigeria; olaitanayinde@gmail.com; 11 Department of Medical Laboratory Sciences, Faculty of Basic Medical Sciences, Redeemer’s University, Ede 232101, Nigeria; osasona23@run.edu.ng; 12 Hospitals Management Board, Ado-Ekiti 360102, Nigeria; 13 National Veterinary Research Institute, Vom 930101, Nigeria; drdammy@gmail.com; 14 Infectious Disease Institute, College of Medicine, University of Ibadan, Ibadan 200212, Nigeria

**Keywords:** human cosavirus, high-throughput sequencing, metagenomics, acute flaccid paralysis, Nigeria

## Abstract

Since its discovery via metagenomics in 2008, human cosavirus (HCoSV) has been detected in the cerebrospinal fluid (CSF) and feces of humans with meningitis, acute flaccid paralysis (AFP), and acute gastroenteritis. To date, 34 HCoSV genotypes have been documented by the *Picornaviridae* study group. However, the documented genetic diversity of HCoSV in Nigeria is limited. Here we describe the genetic diversity of HCoSV in Nigeria using a metagenomics approach. Archived and anonymized fecal specimens from children (under 15 years old) diagnosed with non-polio AFP from five states in Nigeria were analyzed. Virus-like particles were purified from 55 pools (made from 254 samples) using the NetoVIR protocol. Pools were subjected to nucleic acid extraction and metagenomic sequencing. Reads were trimmed and assembled, and contigs classified as HCoSV were subjected to phylogenetic, pairwise identity, recombination analysis, and, when necessary, immuno-informatics and capsid structure prediction. Fifteen pools yielded 23 genomes of HCoSV. Phylogenetic and pairwise identity analysis showed that all belonged to four species (eleven, three, three, and six members of *Cosavirus asiani*, *Cosavirus bepakis*, *Cosavirus depakis*, and *Cosavirus eaustrali*, respectively) and seventeen genotypes. Ten genomes belong to seven (HCoSV-A3/A10, A15, A17, A19, A24, D3, and E1) previously assigned genotypes, while the remaining thirteen genomes belonged to ten newly proposed genotypes across the four HCoSV species, based on the near-complete VP1 region (VP1*) of the cosavirus genome. Our analysis suggests the existence of at least seven and eight *Cosavirus bepakis* and *Cosavirus eaustrali* genotypes, respectively (including those described here). We report the first near-complete genomes of *Cosavirus bepakis* and *Cosavirus depakis* from Nigeria, which contributes to the increasing knowledge of the diversity of HCoSV, raising the number of tentative genotypes from 34 to over 40. Our findings suggest that the genetic diversity of HCoSV might be broader than is currently documented, highlighting the need for enhanced surveillance.

## 1. Introduction

Many virus species and types remain unidentified in clinical and environmental samples due to the low rate of virus identification achieved by conventional methods including Sanger sequencing, polymerase chain reaction (PCR), and virus isolation in cell culture [1]. A huge expansion of newly discovered viruses has been observed in recent times due to the introduction of various Next Generation Sequencing technologies, allowing viral metagenomics and high-throughput sequencing, which have enhanced our understanding of virus diversity in clinical and environmental samples [2,3,4,5,6]. Fecal viromes have been established to be a rich source of novel viruses [7,8,9,10,11,12], including human cosavirus (HCoSV), which was first reported in 2008 from feces of South Asian children with non-polio acute flaccid paralysis (AFP) [13]. Subsequently, HCoSVs have been found in stool samples of humans with acute gastroenteritis (AGE) or AFP [14,15,16,17], environmental samples [18], and cerebrospinal fluid [19].

The pathogenicity of HCoSV remains ambiguous, as it has also been detected in a significant proportion of healthy subjects [13,20]. Further, its frequent co-infections with other viruses at a relatively low prevalence, as well as low viral RNA concentrations in the feces of patients with AFP and gastroenteritis, corroborate this unclear link with various pathologies [13,14,21,22,23]. Disease association studies of HCoSV are made more complex due to its extensive genetic variation, since various virus species and/or genotypes might cause differing clinical symptoms in infected individuals [24].

HCoSVs belong to the genus *Cosavirus*, in the family *Picornaviridae*, and have a positive-sense, single-stranded RNA genome encapsidated within a non-enveloped icosahedral capsid with a diameter of ~30 nm. The genome is 7–8 kb in length, linear and non-segmented, encoding for a single polyprotein, which is subsequently cleaved into P1, P2, and P3 proteins. Further, seven non-structural proteins are produced by the further cleavage of P2 and P3 (2A, 2B, 2C, 3A, 3B, 3C, and 3D), while four structural polypeptides are produced by further cleavage of P1 (VP4, VP2, VP3, and VP1).

The HCoSV genomes detected to date have been grouped into six species (*Cosavirus asiani*, *Cosavirus bepakis,* *Cosavirus depakis*, *Cosavirus eaustrali*, and *Cosavirus fepakis*, formally referred to as *Cosavirus* A–F, respectively; there is no publicly available record yet of a complete genome of species C) and about 34 genotypes based on the ≥88% amino acid similarity in the VP1* (~900 nt spanning the 3’end of VP3 and about 75% of VP1) region [13,18,21,24,25]. Specifically, variants that have >88% amino acid similarity in the VP1* region are considered to belong to the same HCoSV genotype. Though *Cosavirus asiani* and *Cosavirus depakis* are more commonly reported, recombinant (E/D) strains have also been identified [13,18,21,22,24,25]. According to the International Committee on Taxonomy of Viruses (ICTV) species delineation criteria [26], HCoSVs belong to the same species if they have a shared genome architecture and have <35% amino acid divergence in their P1 aa sequence and <10% divergence in their 2C + 3CD aa sequence (https://ictv.global/report/chapter/picornaviridae/picornaviridae/cosavirus, accessed on 25 May 2025).

In this study, we detected 23 HCoSV genomes belonging to 17 genotypes in four species (*Cosavirusasiani*, *Cosavirus bepakis*, *Cosavirus depakis*, and *Cosavirus eaustrali*) in archived and anonymized fecal samples from children with non-polio AFP in Nigeria in the year 2020. We show high genetic diversity of HCoSVs circulating in Nigeria and further our understanding of HCoSV diversity by describing ten tentative new genotypes (three, three, two, and two, respectively inside HCoSV-A, HCoSV-B, HCoSV-D, and HCoSV-E) based on reference sequences provided by the Picornavirus study group https://www.picornaviridae.com/caphthovirinae/cosavirus/cosavirus.htm (accessed on 25 May 2025). Our data therefore suggests an increase in documented HCoSV genotypes from 34 to over 40.

## 2. Methods

### 2.1. Sample Collection and Preparation

The study analyzed archived and anonymized fecal samples obtained from Nigerian children under the age of 15 who were diagnosed with acute flaccid paralysis (AFP) in the year 2020. According to laboratory data, between January and December 2020, a total of 6330 fecal samples from AFP cases were received by the WHO National Polio Laboratory in Ibadan, Nigeria. The fecal samples were obtained in accordance with national ethical guidelines as part of Nigeria’s National AFP monitoring initiative and transported to the Polio Laboratory. The WHO algorithm, as outlined in the WHO Polio Manual [27], was utilized to ascertain if poliovirus was the cause of the detected cases of AFP. Specifically, 254 non-polio AFP fecal samples collected from five regions (the Federal Capital Territory, Lagos, Kaduna, Anambra, and Edo) between January and December 2020 were randomly chosen for inclusion in this study. Prior to analysis, the samples were anonymized and then grouped by their respective states of origin and collection months. Each fecal sample (approximately 0.5 g) was diluted in phosphate-buffered saline (PBS; 4.5 mL) mixed with glass beads (0.5 g) before 20 min vortexing followed by 20 min centrifugation at 1469× *g*. Two cryovials, one for each of the two mL of the resultant supernatant, were aliquoted and kept at −20 °C. To create sample pools, 200 μL of each fecal suspension were mixed. The number of samples in each pool ranged from two to seven, except for one that contained only one fecal suspension. In total, fifty-five pools were obtained from the 254 fecal samples and were subsequently transported on dry ice to the Laboratory of Viral Metagenomics at Katholieke Universiteit Leuven in Belgium.

### 2.2. Nucleic Acid Extraction, PCR Amplification, Library Preparation, and Sequencing

The Novel Enrichment Technique of VIRomes (NetoVIR) [8]. was employed to analyze the fecal suspension. After passing through a 0.8 µm PES filter (Sartorius, Goettingen, Germany), the fecal suspensions were treated with a mixture of Benzonase (Millipore, Billerica, MA, USA), and Micrococcal Nuclease (New England Biolabs, Ipswich, MA, USA) at 37 °C for two hours. Nucleic acid extraction was then carried out using the QIAamp Viral RNA Mini Kit (Qiagen, Hilden, Germany) following the manufacturer’s instructions. Subsequent steps included first and second strand synthesis, along with random PCR amplification for 17 cycles using the Whole Transcriptome Amplification (WTA2) Kit (Sigma-Aldrich, St. Louis, MO, USA) with slight modifications. Purification of the amplified products was conducted using the MSB Spin PCRapace purification kit (Stratec Biomedical, Birkenfeld, Germany). For library preparation for Illumina sequencing (Illumina, San Diego, CA, USA), the Nextera XT Library Preparation Kit was used with slight modifications. Sequencing of the samples was performed in paired-end mode (2 × 150 bp) using an Illumina Novaseq 6000 platform.

### 2.3. Sequence Assembly

The raw reads were processed using the Virome Paired-End Reads (ViPER) pipeline, available at https://github.com/Matthijnssenslab/ViPER. Bowtie 2 was employed to filter out reads aligned with the human genome, while Trimmomatic was utilized to trim the reads to ensure high quality [28]. Subsequently, the reads were *de novo* assembled into contigs using metaSPAdes [29]. The assembled contigs were then annotated using DIAMOND’s sensitive option [30]. Contigs of interest were named in the following format: node ID_sample-pool_country_year. For example, NODE_A19_AFP6_NGR_2020.

### 2.4. Virus Typing and Recombination Analysis

Virus typing involved utilizing a combination of methods like BLASTn searches of the GenBank database, reference sequences sourced from the Picornavirus study group [31] and ICTV HCoSV page [26], as well as phylogenetic and pairwise identity analyses. Genomes identified in this investigation served as queries in a BLASTn search conducted on the GenBank database. The top matches were retrieved and incorporated into a local database (referred to as database 1), alongside the HCoSV contigs obtained in this study. Additionally, reference sequences from the Picornavirus study group [31] and the ICTV HCoSV page [26] were downloaded, deduplicated, and included in a second local database (database 2), alongside the HCoSV contigs from this study. It is germane to note that while database 1 predominantly contained complete or nearly complete genomes, database 2 mainly consisted of smaller sequences (specifically the VP1* region; approximately 900 nucleotides spanning the 3′ end of VP3 and about 75% of VP1 [24]).

Database 2 contains numerous HCoSV genotypes that are absent in Database 1. Consequently, for analyses that required complete coding sequences (such as species designation and recombination analysis), Database 1 was utilized. Conversely, for analyses requiring only the VP1 region, like genotype designation, either Database 2 or a merged version with deduplication were employed. The selected database was subjected to multiple sequence alignment (MSA) utilizing the MUSCLE algorithm, and phylogenetic trees were constructed using the maximum likelihood algorithm (Tamura-Nei model, 1000 bootstrap) in MEGA X [32]. Pairwise identity was calculated using SDT v1.2 [33]. Simplot and Bootscan analyses were conducted using sequences from Database 1 in SimPlot v3.5.1 [34]. HCoSV contigs and mapped reads generated in this study have been submitted to GenBank and SRA under accession numbers PP386510-PP386532 and PRJNA107798, respectively.

### 2.5. Immuno-Informatic and Structural Analysis

B-Cell epitopes were predicted using the Bepipred Linear Epitope Prediction 2.0 [35]. HCoSV protomers were predicted using alphafold2 as implemented in ColabFold [36]. The complete HCoSV capsid was predicted using the oligomer generator in Viperdb [37]. Structure alignment and annotation were conducted in ChimeraX v1.8 [38].

## 3. Results

### 3.1. HCoSV Detection and Genotyping

**Figure 1:**
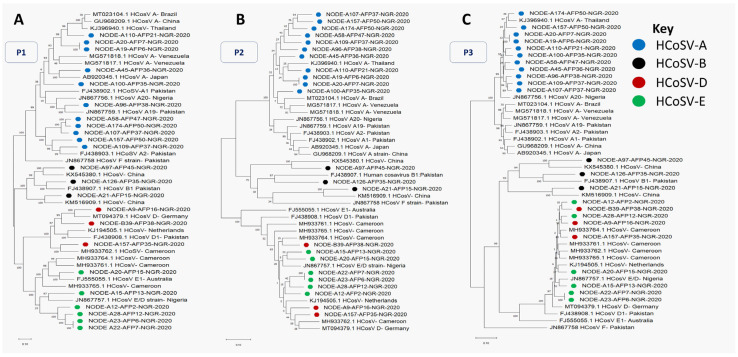
Maximum likelihood phylogenetic trees of HCoSV variants described in this study alongside some reference sequences. Analysis was conducted using nucleotide sequences of the complete (**A**) P1, (**B**) P2, and (**C**) P3 genomic regions.

**Figure 2:**
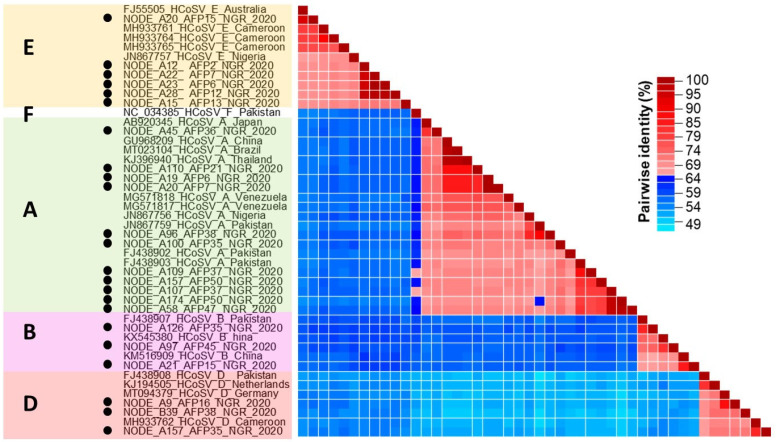
Pairwise identity analysis of HCoSV variants described in this study alongside some reference sequences. The letters on the left side of the image are the different HCoSV species. Species boundaries are demarcated with different colors. Analysis was conducted using the complete P1 amino acid sequence. Sequences detected in this study are noted with black circles. Please note that sequence IDs at the bottom of the matrix have been removed to enhance visibility.

Non-polio fecal samples (*n* = 254) collected from five regions in Nigeria between January and December 2020 and grouped into 55 pools were analyzed for this study. Twenty-three (23) HCoSV genomic contigs were obtained from the samples analyzed. These include 20 near-complete genome sequences and three partial genome sequences, as listed in Table 1. A BLASTn search shows that eleven, three, three, and six of these contigs are members of *Cosavirus asiani* (HCoSV-A), *Cosavirus bepakis* (HCoSV-B), *Cosavirus depakis* (HCoSV-D), and *Cosavirus eaustrali* (HCoSV-E), respectively (Table 1). This was validated by phylogenetic and pairwise identity analysis of the P1 genomic region (Figure 1 and Figure 2). The 23 HCoSV contigs detected in this study were obtained from 15 of the 55 pools analyzed (Table 2). The number of contigs per sample pool varied from zero to three (Table 2).

**Figure 3:**
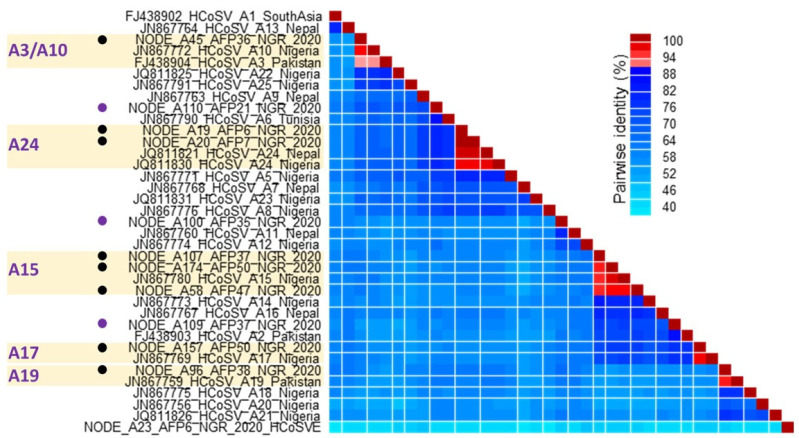
Pairwise identity analysis of HCoSV-A variants described in this study alongside some reference sequences. Analysis was conducted using the VP1* amino acid sequence as described in [24]. The eleven HCoSV-A variants detected in this study belong to eight genotypes; five previously assigned genotypes (black circles) and three tentative new ones (purple circles). The letters on the left side of the image are different HCoSV-A genotypes that belong to the five previously assigned genotypes (black circles). Genotype boundaries are indicated with color. Please note that sequence IDs at the bottom of the matrix have been removed to enhance visibility.

Analysis of pairwise identity for VP1* revealed that the 11 HCoSV-A variants identified in this study belong to 8 distinct genotypes (Figure 3). Five of the eight genotypes include HCoSV-A3/A10, A15, A17, A19, and A24. The remaining three strains show pairwise identity values that are below the cut-off (88% amino acid) and may therefore be representatives of novel genotypes (Figure 3).

**Figure 4:**
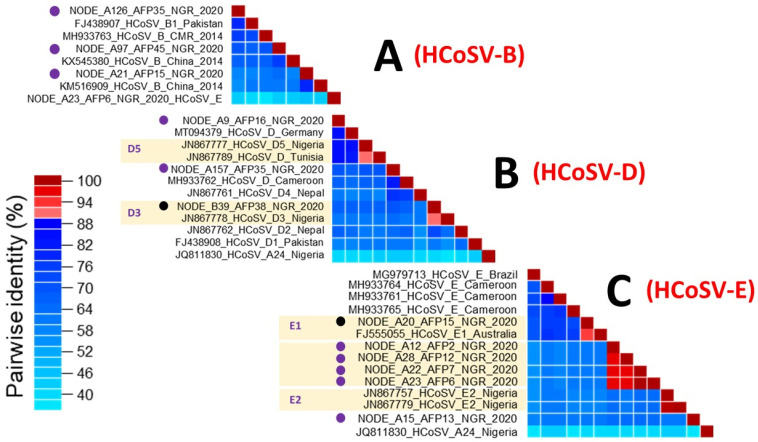
Pairwise identity analysis of HCoSV-B (**A**), D (**B**), and E (**C**) variants described in this study alongside some reference sequences. Analysis was conducted using the VP1* amino acid sequence as described in [24]. The 12 HCoSV-B, D, and E variants detected in this study are indicated with circles. Black circles denote variants belonging to genotypes that had been previously assigned. Purple circles denote variants belonging to tentative new genotypes. Please note that sequence IDs at the bottom of the matrix have been removed to enhance visibility.

Three variants each of HCoSV-B and HCoSV-D, were detected. However, three of the HCoSV-B variants and two of the HCoSV-D variants detected in this study might be novel genotypes due to pairwise identity values that are below 88% with the established genotypes (Figure 4A,B). The third HCoSV-D variant was successfully identified as a member of D3 (Figure 4B). Though only one reference HCoSV-B type (B1) seems to have been assigned https://www.picornaviridae.com/caphthovirinae/cosavirus/cosavirus_b/cosavirus_b.htm (accessed on 25 May 2025), our analysis based on publicly available data suggests that at least seven distinct HCoSV-B genotypes have been detected globally (Figure 4A). Similarly, only five of the nine HCoSV-D genotypes detected globally to date (Figure 4B) have been assigned genotypes https://www.picornaviridae.com/caphthovirinae/cosavirus/cosavirus_d/cosavirus_d.htm (accessed on 25 May 2025). 

For HCoSV-E, six variants were detected. Pairwise identity analysis identified one of the variants as a member of HCoSV-E1. The remaining five were, however, typed as members of two tentative new genotypes with one and four members each (Figure 4C). Though only two reference HCoSV-E genotypes (E1 and E2) have been assigned https://www.picornaviridae.com/caphthovirinae/cosavirus/cosavirus_e/cosavirus_e.htm (accessed on 25 May 2025), our analysis based on publicly available data suggests that at least eight distinct HCoSV-E genotypes have been detected globally (Figure 4).

In summary, fifteen pools yielded 23 variants of HCoSV that belong to four species (eleven, three, three, and six members of HCoSV A, B, D, and E, respectively) and 17 genotypes. Ten variants belong to seven (HCoSV-A3/A10, A15, A17, A19, A24, D3, and E1) previously assigned genotypes, while the remaining thirteen variants belong to ten tentative new genotypes in four HCoSV species. Our analysis suggests at least seven and eight B and E genotypes, respectively (including those described in this study), have been described globally to date but are yet to be assigned to genotypes. Furthermore, HCoSV genotypes A3 and A10 might refer to the same virus type.

### 3.2. Recombination Analysis

**Figure 5:**
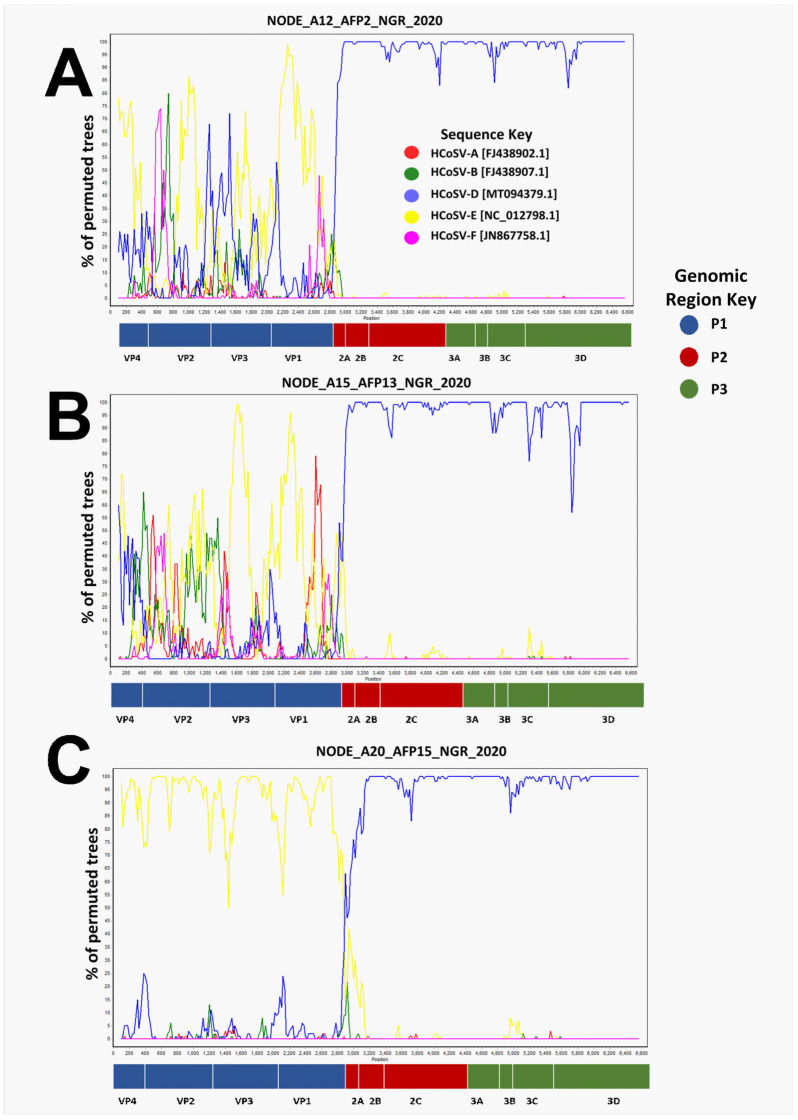

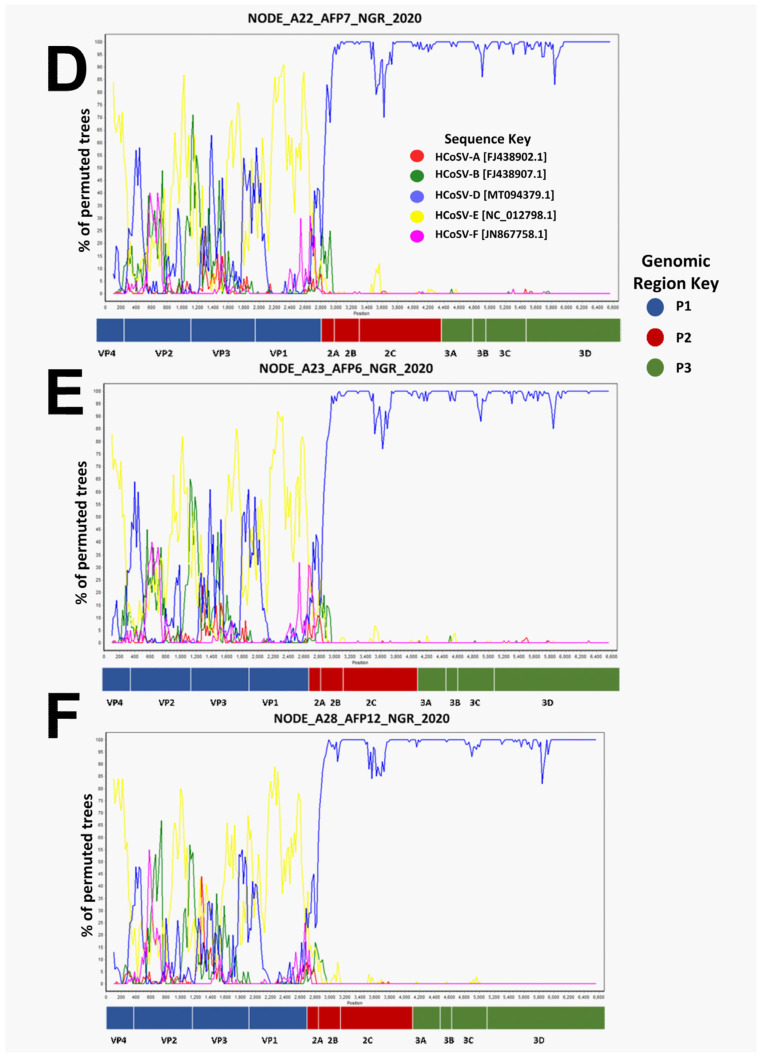
Bootscan analysis showing recombination events in the HCoSV-E sequences. Analysis was conducted using the suspected recombinant sequences as queries (**A**–**F**) against reference HCoSV species HCoSV-A, HCoSV-B, HCoSV-D, HCoSV-E and HCoSV-F. A single breakpoint between P1 and P2 genomic regions was observed.

Phylogenetic analysis of P1, P2, and P3 genomic regions showed phylogeny violations, suggesting there might be ongoing recombination between members of HCoSV-D and HCoSV-E (Figure 1). This was confirmed using Bootscan analysis. A single breakpoint was detected, generally at the P1/P2 junction (Figure 5). To rule out the likelihood that the detected recombination could be an artifact of pooling samples, we examined instances of co-detection of HCoSV-D and HCoSV-E in the pools and noticed that none of the pools included both HCoSV-D and HCoSV-E (Table 2).

### 3.3. Immuno-Informatic and Structural Analysis

**Figure 6:**
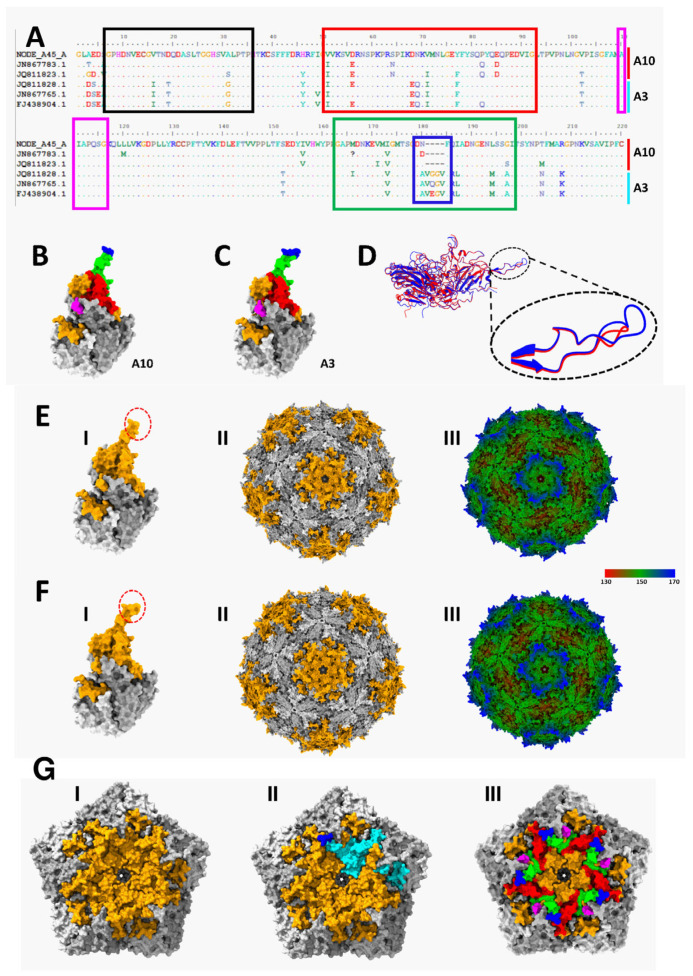
Amino acid and structural differences between A10 (NODE_A45_AFP36_NGR_2020) and A3 (FJ438904) (**A**) show an alignment of the amino acid translation of the VP1 region of the VP1* amplicon of six sequences (three each of A3 and A10). Boxes labeled black, red, magenta, and green highlight predicted B-cell epitopes. The blue box within ‘green’ highlights a 4-amino acid insertion in A3 that is absent in A10. (**B**,**C**) show predicted structures of A10 and A3 protomers, respectively, with predicted surface-exposed B-cell epitopes in A layered onto them. Please note that the predicted B-cell epitope highlighted in black is not surface exposed. In (**B**,**C**,**EI**,**EII**,**FI**,**FII**), capsid proteins VP1, VP2, and VP3 are labeled orange, dark grey, and light grey, respectively. (**D**) shows structural alignment of B (A10) and C (A3) in ribbon model and the inset highlights the beta-loop where the 4-amino acid insertion is located. EI and EII show predicted structures of A10 protomer and capsid, respectively. (**EIII**) shows predicted structure of A10 capsid radially colored. (**FI**,**II**) show predicted structures of A3 protomer and capsid, respectively. (**FIII**) shows predicted structure of A3 capsid radially colored. (**GI**–**III**) show closeup of 5-fold axis of symmetry in (**EII**). In (**GII**) one VP1 is colored and the beta-loop highlighted in blue. In (**GIII**), VP1 is labeled as in (**B**).

We further explored the A3/A10 similarity conundrum. Multiple sequence alignment of the VP1 region of VP1* of A3 and A10 sequences showed some amino acid conservation between both (Figure 6A). There were four predicted B-cell epitopes highlighted with black, red, magenta, and green boxes. In the predicted B-cell epitope labelled green, there is a four-amino acid insertion which seems to be a defining feature of A3. Protomer prediction (Figure 6B–D) showed that the four-amino acid insertion is within a β-loop. Capsid prediction showed that the four-amino acid insertion on five protomers is around the rim of the wall surrounding the predicted five-fold axis of symmetry (Figure 6E–G).

## 4. Discussion

In this study, using a metagenomic approach, we detected 23 HCoSV variants belonging to 17 genotypes in four species (eleven, three, three, and six members of HCoSV A, B, D, and E, respectively) in archived and anonymized fecal samples from children with non-polio AFP in Nigeria in the year 2020. We show high genetic diversity of HCoSVs circulating in Nigeria and further expand our understanding of HCoSV diversity by describing ten tentative new genotypes (three, three, two, and two of HCoSV-A, HCoSV-B, HCoSV-D, and HCoSV-E, respectively) based on refseqs provided by the picornavirus study group [31]. Our data therefore suggests an increase in documented HCoSV genotypes from 34 to over 40.

Overall, HCoSV-A species accounted for approximately 50% (11 out of 23) of all HCoSV genomes identified in this study. These results corroborate previous research indicating the prevalent presence of HCoSV-A compared to other species in clinical samples [14,16,17,19,20,23,24,39,40,41,42]. Though 25 HCoSV-A genotypes have been previously assigned (https://www.picornaviridae.com/caphthovirinae/cosavirus/cosavirus_a/cosavirus_a.htm (accessed on 25 May 2025)), 8 different HCoSV-A genotypes have been identified from this study, of which 3 are tentative new genotypes (Figure 3). The widespread distribution of HCoSV-A species members could account for their genetic diversity (HCoSV-A1 to A25 as of May 2025) relative to other species. Pairwise identity analysis (Figure 3) showed that A3 and A10 had high similarity of ≥88% (aa), suggesting they likely belong to the same genotype. Furthermore, based on conservation in the other amino acid residues in the predicted B-cell epitopes in VP1* of A3 and A10 (Figure 6A), relative to those of the other HCoSV-As (Figure S1) A3 and A10 might have to be reconsidered as the same genotype.

A search of the protein databank for any solved structure of HCoSV capsid was not productive. Consequently, we predicted the structure of the HCoSV capsid and layered predicted VP1 B-cell epitopes onto it (Figure 6). It is important to note that the topology of the predicted 5-fold axis of symmetry coupled with the clustering of predicted B-cell epitopes suggests that antibodies that bind there will likely sterically occlude access to the center of the 5-fold axis of symmetry (Figure 6G). A3, however, has a unique four-amino acid insertion in one of the predicted antigenic β-loops (Figure 6A–D). Considering the location of this protrusion on the outer bounds of the wall surrounding the five-fold axis of symmetry (Figure 6G), it is not clear how the four-amino acid insertion might impact host range, receptor usage, serology, and/or other phenotypes of the virus. However, the fact that one of the four amino acids is evolving (Figure 6A) suggests it might be under evolutionary pressure. Hence, antibodies binding to this site might sterically occlude access to other sites (possibly the surface depression) around the five-fold axis of symmetry (Figure 6(FIII)).

For the first time in Nigeria, we report three near-complete HCoSV B genomes. While previous instances of HCoSV B have been documented among patients with AFP and gastroenteritis in Cameroon, China, and Pakistan [13,16,43], none has been documented in Nigeria until now. According to the phylogenetic analysis, the three HCoSV B genomes formed a cluster with reference B strains (Figure 1). However, pairwise identity analysis showed that none of the three sequences clustered with the single previously assigned genotype (B1), suggesting the novelty of the B variants detected in this study (Figure 4). Further, the pairwise identity analysis based on publicly available data suggests that at least seven distinct HCoSV-B genotypes have been detected globally (Figure 4A). This study further supports the suggestion that the diversity of HCoSV-B might be broader than has been reported [44].

Three different HCoSV-D variants were detected in this study, of which one belongs to a previously assigned (D3) type (Figure 4). A partial genome of D3 had been previously reported from Nigeria (Figure 4B) in 2007 [24]. Though the HCoSV-D strain was recently detected in healthy persons from Nigeria using a 5′-UTR sequence [20], this report represents the first near-complete HCoSV-D genomes from Nigeria and further demonstrates that the diversity of HCoSV-D might also be broader than has been reported.

Positive-sense single-stranded RNA viruses, such as HCoSVs, prominently demonstrate high rates of recombination [45,46]. A Nigerian AFP patient was the source of the first report of the recombination pattern of HCoSVs at the P1/P2 junction in 2012 (strain NG385; JN867757). Subsequent detections also include a clinical case in a Brazilian patient with acute gastroenteritis in 2018 and identification in asymptomatic children from Venezuela in 2015 (strain BRTO-83) [22,24,47]. We now report the sequencing of six (6) nearly complete genomic sequences of recombinant (E/D) HCoSV strains from 2020 Nigerian AFP samples that were poliovirus negative. Phylogenetic analysis and pairwise amino acid identity comparisons between the six genomes and reference genomes of species A-F from GenBank revealed that the six genomes shared greater similarity with species E in the P1 region (>65%) (Figure 1 and Figure 2), whereas they were more closely related to species D in the P2 and P3 genomic regions (Figure 1). Furthermore, Bootscan analysis substantiates the occurrence of a recombination event at the P1/P2 junction (Figure 5). This pattern mirrors the initial observation of HCoSV recombination in the Nigerian AFP sample [24], thus making this study a second report of HCoSV recombinant E/D strains in Nigeria. Following the VP1* pairwise identity analysis, one of the six variants of HCoSV-E (Figure 1 and Figure 2) was identified as a member of HCoSV-E1 (≥88% aa similarity), while the remaining five were found to belong to two tentative new genotypes with one and four members each (Figure 4). Hence suggesting HCoSV-E might also be more diverse than currently documented.

Though the detection of HCoSV is increasing, especially in association with AFP and gastroenteritis, further surveillance and molecular epidemiological investigations are necessary to better understand the circulation, distribution, and evolutionary dynamics of HCoSV genotypes globally. Also, virus isolation in cell culture might be essential, as it will enhance our understanding of genotype-to-serotype boundaries of HCoSVs and consequently facilitate investigations into the probable etiological potential of the various HCoSV genotypes.

## Figures and Tables

**Table 1:** BLASTn results of HCoSV genomic sequences detected in this study and those most similar to them in GenBank.

Contig ID	Genome Length	Coding Sequence	Mean Depth of Coverage	Species	Accession; Query Cover (%); Pairwise Identity (%); Country; Year	
					Best Hit 1	Best Hit 2
NODE_A12_AFP2_NGR_2020	7527	Complete	51×	HCoSV E	JN867757.1; 77; 91.1; Nigeria; 2007	MH933764.1;77: 90.73; Cameroon; 2014
NODE_A19_AFP6_NGR_2020	7761	Complete	464×	HCoSV A	MG571817.1; 94; 88.3; Venezuela; 2015	MG571823.1; 94; 84.1; VENEZUELA; 2015
NODE_A23_AFP6_NGR_2020	6662	Complete	131×	HCoSV E	MG571820; 74; 83.9; Venezuela; 2015	MH933764.1; 66; 91; Cameroon; 2014
NODE_A20_AFP7_NGR_2020	7785	Complete	288×	HCoSV A	MG571817.1;94;88.3; Venezuela; 2015	MG571823.1; 94; 84.1; VENEZUELA; 2015
NODE_A22_AFP7_NGR_2020	7475	Complete	80×	HCoSV E	MG571820; 77; 83.9; Venezuela; 2015	MH933764.1; 69; 90.9 Cameroon; 2014
NODE_A28_AFP12_NGR_2020	7490	Complete	138×	HCoSV E	JN867757.1; 77; 91.1; Nigeria; 2007	MH933764.1; 69; 92.3 Cameroon; 2014
NODE_A15_AFP13_NGR_2020	7763	Complete	1029×	HCoSV E	JN867757.1; 76; 91.5; Nigeria; 2007	MH933764.1; 71; 90.3 Cameroon; 2014
NODE_A20_AFP15_NGR_2020	7194	Complete	222×	HCoSV E	MH933764.1; 100; 84.8; Cameroon; 2014	MH933765.1; 100; 83.74; Cameroon; 2014
NODE_A21_AFP15_NGR_2020	7149	Complete	945×	HCoSV B	KM516909.1;99;79.4; China; 2012	GU968209.1; 28; 84.51; China; 2009
NODE_A9_AFP16_NGR_2020	6610	Complete	80×	HCoSV D	MT094379.1; 99; 84.6; Germany; 2018	MH933762.1; 72; 87.5;Cameroon; 2014
NODE_A110_AFP21_NGR_2020	7678	Complete	78×	HCoSV A	MG571817; 97; 88.3; Venezuela; 2015	MG571823.1; 95; 84.1; Venezuela; 2015
NODE_A100_AFP35_NGR_2020	7747	Complete	264×	HCoSV A	MG571817; 85; 88.1; Venezuela; 2015	FJ438903.1; 81; 87.4; Pakistan
NODE_A126_AFP35_NGR_2020	6723	Complete	38×	HCoSV B	FJ438907; 99; 74.8; Pakistan	MH933763.1; 38; 72.3; Cameroon; 2014
NODE_A157_AFP35_NGR_2020	5983	Partial	27×	HCoSV D	MH933762.1; 98; 85.2; Cameroon; 2014	MT094379.1; 93; 87.8; Germany; 2018
NODE_A45_AFP36_NGR_2020	7794	Complete	320×	HCoSV A	AB920345; 93; 82.6; Japan; 2012	FJ438904.1: 82: 83.81; Pakistan
NODE_A107_AFP37_NGR_2020	6668	Complete	369×	HCoSV A	FJ438903; 95; 87.6; Pakistan	KJ396940; 85; 90.6; Thailand; 2011
NODE_A109_AFP37_NGR_2020	6613	Complete	181×	HCoSV A	FJ438903; 99; 83.1; Pakistan	MG571817.1; 89; 86.5; Venezuela; 2015
NODE_A96_AFP38_NGR_2020	7388	Complete	300×	HCoSV A	JN867759; 89; 84.7; Pakistan; 2006	GU968209; 87; 84.8; China; 2009
NODE_B39_AFP38_NGR_2020	6735	Complete	369×	HCoSV D	MT094379.1; 96; 87.3; Germany; 2018	KJ194505.1; 86; 90.4; Netherlands; 1994
NODE_A97_AFP45_NGR_2020	6618	Partial	1097×	HCoSV B	KX545380; 97; 76.1; China; 2014	MH933763; 50; 72.4;Cameroon; 2014
NODE_A58_AFP47_NGR_2020	7756	Complete	216×	HCoSV A	FJ438903; 94; 82.9; Pakistan	MT023104.1; 82; 87.6; Brazil; 2017
NODE_A157_AFP50_NGR_2020	5257	Partial	135×	HCoSV A	FJ438903; 99; 79.6; Pakistan	MG571818.1; 71; 85.6; Venezuela; 2015
NODE_A174_AFP50_NGR_2020	4931	Partial	141×	HCoSV A	FJ438903; 99; 79; Pakistan	GU968209.1; 79; 84.7; China; 2009

**Table 2:** HCoSV genotype distribution by AFP sample pools.

S/N	Sample Pool ID	Number of Samples in Pool	Number of Variants	HCoSV Genotype(s)
1	AFP2	6	1	E-New
2	AFP6	5	2	A24, E-New
3	AFP7	5	2	A24, E-New
4	AFP12	1	1	E-New
5	AFP13	5	1	E-New
6	AFP15	7	2	B-New, E1
7	AFP16	5	1	D-New
8	AFP21	5	1	A-New
9	AFP35	5	3	A-New, B-New, D-New
10	AFP36	4	1	A3/A10
11	AFP37	5	2	A-New, A15
12	AFP38	5	2	A19, D3
13	AFP45	2	1	B-New
14	AFP47	2	1	A15
15	AFP50	5	2	A15, A17
	Total	67	23	17 (including 10 new genotypes)

## Data Availability

The datasets generated and/or analyzed during the current study are available in GenBank and SRA under accession numbers PP386510-PP386532 (https://www.ncbi.nlm.nih.gov/nuccore/PP386510) and PRJNA107798, respectively.

## Supplementary Materials

The following supporting information can be downloaded at: https://www.mdpi.com/article/10.3390/v17060844/s1, Figure S1: Alignment showing the amino acid translation of only selected (red, magenta and green) predicted B-cell epitopes in the VP1 region of the VP1*. This alignment includes reference sequences of all currently typed HCoSV-As alongside those described in this study. NODE_A45_AFP36_NGR_2020 (A10) and FJ438904 (A3) are sequences 1 and 2, respectively in the alignment.

## References

[1] Woolhouse M.F., Scott Z., Hudson R., Howey M. (2012). Chase-Topping. Human viruses: Discovery and emergence. Philos. Trans. R. Soc. B Biol. Sci..

[2] Wang W.J., Jovel B., Halloran E., Wine J., Patterson G., Ford S., O’Keefe B., Meng D., Song Y., Zhang Z. (2015). Metagenomic analysis of microbiome in colon tissue from subjects with inflammatory bowel diseases reveals interplay of viruses and bacteria. Inflamm. Bowel Dis..

[3] Osunmakinde C.O., Selvarajan R., Sibanda T., Mamba B.B., Msagati T.A.M. (2018). Overview of trends in the application of metagenomic techniques in the analysis of human enteric viral diversity in Africa’s environmental regimes. Viruses.

[4] Deaton J., Yu F.B., Quake S.R. (2019). Mini-metagenomics and nucleotide composition aid the identification and host association of novel bacteriophage sequences. Adv. Biosyst..

[5] Hamza I.A., Bibby K. (2019). Critical issues in application of molecular methods to environmental virology. J. Virol. Methods.

[6] Li R., Zhu L., Cui L., Zhu Y.G. (2022). Viral diversity and potential environmental risk in microplastic at watershed scale: Evidence from metagenomic analysis of plastisphere. Environ. Int..

[7] Finkbeiner S.R., Allred A.F., Tarr P.I., Klein E.J., Kirkwood C.D., Wang D. (2008). Metagenomic analysis of human diarrhea: Viral detection and discovery. PLoS Pathog..

[8] Conceicao-Neto N., Zeller H., Lefrere P., De-Bruyn L., Beller W., Deboutte C.K., Yinda R., Lavigne P., Maes M., Van-Ranst E. (2015). Modular approach to customise sample preparation procedures for viral metagenomics: A reproducible protocol for virome analysis. Sci. Rep..

[9] Lojkic I., Bidin M., Prpic J., Simic I., Kresic N., Bedekovic T. (2016). Faecal virome of red foxes from peri-urban areas. Comp. Immunol. Microbiol. Infect. Dis..

[10] Lima D.A., Cibulski S.P., Finkler F., Teixeira T.F., Varela A.P.M., Cerva C., Loiko M.R., Scheffer C.M., Dos Santos H.F., Mayer F.Q. (2017). Faecal virome of healthy chickens reveals a large diversity of the eukaryote viral community, including novel circular ssDNA viruses. J. Gen. Virol..

[11] Duarte M.A., Silva J.M.F., Brito C.R., Teixeira D.S., Melo F.L., Ribeiro B., Nagata T., Campos F.S. (2019). Faecal virome analysis of wild animals from Brazil. Viruses.

[12] Lima D.A., Cibulski S.P., Tochetto C., Varela A.P.M., Finkler F., Teixeira T.F., Loiko M.R., Cerva C., Junqueira D.M., Mayer F.Q. (2019). The intestinal virome of malabsorption syndrome-affected and unaffected broilers through shotgun metagenomics. Virus Res..

[13] Kapoor A., Victoria J., Simmonds P., Slikas E., Chieochansin T., Naeem A., Shaukat S., Sharif S., Alam M.M., Angez M. (2008). A highly prevalent and genetically diversified picornaviridae genus in south asian children. Proc. Natl. Acad. Sci. USA.

[14] Stöcker A., Breno F., de Carvalho D., Souza T., Cristina M., Ribeiro E., Martins N., Luciana O., Araujo J., Ivan C. (2012). Cosavirus infection in persons with and without gastroenteritis, Brazil. Emerg. Infect. Dis..

[15] Okitsu S., Khamrin P., Thongprachum A., Nishimura S., Kalesaran A.F.C., Takanashi S., Shimizu H., Hayakawa S., Mizuguchi M., Ushijima H. (2014). Detection and molecular characterization of human cosavirus in a pediatric patient with acute gastroenteritis, Japan. Infect. Genet. Evol..

[16] Yang Y., Ju A., Xu X., Cao X., Tao Y. (2016). A novel type of cosavirus from children with nonpolio acute flaccid paralysis. Virol. J..

[17] Vizzi E.R., Fernández L.A., Angulo R., Blanco, Pérez C. (2021). Human cosavirus infection in HIV subjects with diarrhoea: Persistent detection associated with fatal outcome. J. Clin. Virol..

[18] Blinkova O., Rosario K., Li L., Kapoor A., Slikas B., Bernardin F., Breitbart M., Delwart E. (2009). Frequent detection of highly 423 diverse variants of cardiovirus, cosavirus, bocavirus, and circovirus in sewage samples collected in the United States. J. Clin. Microbiol..

[19] Moghaddam F.S., Ghaderi M., Parsania M., Mozhgani S.-H., Arjmand R. (2021). First human cosavirus detection from cerebrospinal fluid in hospitalized children with aseptic meningitis and encephalitis in Iran. Pediatr. Infect. Dis. J..

[20] Osundare F.A., Opaleye O.O., Akindele A.A., Adedokun S.A., Akanbi O.A., Bock C.T., Diedrich S., Böttcher S. (2019). Detection and characterization of human enteroviruses, human cosaviruses, and a new human parechovirus type in healthy individuals in Osun State, Nigeria, 2016/2017. Viruses.

[21] Holtz L.R., Finkbeiner S.R., Kirkwood C.D., Wang D. (2008). Identification of a novel picornavirus related to cosaviruses in a child with acute diarrhea. Virol. J..

[22] Da Costa A.C., Luchs A., de Pádua Milagres F.A., Komninakis S.V., Gill D.E., Lobato M.C.A.B.S., Brustulin R., das Chagas R.T., Dos Santos M.d.F.N., Soares C.V.d.D.A. (2018). Near full length genome of a recombinant (e/d) cosavirus strain from a rural area in the central region of Brazil. Sci. Rep..

[23] Daprà V., Galliano I., Montanari P., Zaniol E., Calvi C., Alliaudi C., Bergallo M. (2021). Bufavirus, cosavirus, and salivirus in diarrheal Italian infants. Intervirology.

[24] Kapusinszky B., Phan T.G., Kapoor A., Delwart E. (2012). Genetic diversity of the genus cosavirus in the family picornaviridae: A new species, recombination, and 26 new genotypes. PLoS ONE.

[25] Cotten M., Oude Munnink B., Canuti M., Deijs M., Watson S.J., Kellam P., van der Hoek L. (2014). Full genome virus detection in fecal samples using sensitive nucleic acid preparation, deep sequencing, and a novel iterative sequence classification algorithm. PLoS ONE.

[26] (2023). International Committee for Taxonomy of Viruses. https://ictv.global/report/chapter/picornaviridae/picornaviridae/cosavirus.

[27] World Health Organization (2004). Polio Laboratory Manual.

[28] Langmead B., Salzberg S.L. (2012). Fast gapped-read alignment with Bowtie 2. Nat. Methods.

[29] Nurk S., Meleshko D., Korobeynikov A., Pevzner P.A. (2017). MetaSPAdes: A new versatile metagenomic assembler. Genome Res..

[30] Buchfink B., Xie C., Huson D.H. (2014). Fast and sensitive protein alignment using DIAMOND. Nat. Methods.

[31] Cosavirus in the Picornavirus Pages. https://www.picornaviridae.com/caphthovirinae/cosavirus/cosavirus.htm.

[32] Kumar S., Stecher G., Li M., Knyaz C., Tamura K. (2018). MEGA X: Molecular Evolutionary Genetics Analysis across Computing Platforms. Mol. Biol. Evol..

[33] Muhire B.M., Varsani A., Martin D.P. (2014). SDT: A virus classification tool based on pairwise sequence alignment and identity calculation. PLoS ONE.

[34] Lole K.S., Bollinger R.C., Paranjape R.S., Gadkari D., Kulkarni S.S., Novak N.G., Ingersoll R., Sheppard H.W., Ray S.C. (1999). Full-length human immunodeficiency virus type 1 genomes from subtype C-infected seroconverters in India, with evidence of intersubtype recombination. J. Virol..

[35] Jespersen M.C., Peters B., Nielsen M., Marcatili P. (2017). BepiPred-2.0: Improving sequence-based B-cell epitope prediction using conformational epitopes. Nucleic Acids Res..

[36] Mirdita M., Schütze K., Moriwaki Y., Heo L., Ovchinnikov S., Steinegger M. (2022). ColabFold: Making protein folding accessible to all. Nat. Methods.

[37] Montiel G.D., Santoyo R.N., Ho P., Carrillo T.M., Iii C.L.B., Johnson J.E., Reddy V.S. (2021). VIPERdb v3.0: A structure-based data analytics platform for viral capsids. Nucleic Acids Res..

[38] Meng E.C., Goddard T.D., Pettersen E.F., Couch G.S., Pearson Z.J., Morris J.H., Ferrin T.E. (2023). UCSF ChimeraX: Tools for structure building and analysis. Protein Sci..

[39] Lobo P.S., Cardoso J.F., Barata R.R., Lemos P.S., Guerra S.F., Soares L.S., Nunes M.R., Mascarenhas J.D. (2020). Near-complete genome of cosavirus A from a child hospitalized with acute gastroenteritis, Brazil. Infect. Genet. Evol..

[40] Okitsu S., Khamrin P., Hanaoka N., Thongprachum A., Takanashi S., Fujimoto T., Mizuguchi M., Shimizu H., Hayakawa S., Maneekarn N. (2016). Cosavirus (family *Picornaviridae*) in pigs in Thailand and Japan. Arch. Virol..

[41] López G.R., Martinez L.M., Freyre L., Freire M.C., Vladimirsky S., Rabossi A., Cisterna D.M. (2021). Persistent Detection of Cosavirus and SaffoldCardiovirus in Riachuelo River, Argentina. Food Environ. Virol..

[42] Schneider J., Engler M., Hofmann J., Selinka H., Jones T., Drosten C., Diedrich S., Corman V., Böttcher S. (2021). Molecular detection of cosaviruses in a patient with acute flaccid paralysis and in sewage samples in Germany. Virus Res..

[43] Yu J.M., Ao Y.Y., Li L.L., Duan Z.J. (2017). Identification of a novel cosavirus species in faeces of children and its relationship with acute gastroenteritis in China. Clin. Microbiol. Infect..

[44] Rezig D., Lamari A., Touzi H., Meddeb Z., Triki H. (2020). Typing of Human Cosaviruses by sequencing of full VP1: Update on global genetic diversity and identification of possible new genotypes circulating in Tunisia, North Africa. Infect. Genet. Evol..

[45] Wang H., Cui X., Cai X., An T. (2022). Recombination in positive-strand RNA viruses. Front. Microbiol..

[46] Simon-Loriere E., Holmes E.C. (2011). Why do RNA viruses recombine?. Nat. Rev. Microbiol..

[47] Siqueira J.D., Dominguez-Bello M.G., Contreras M., Lander O., Caballero-Arias H., Deng X., Noya-Alarcon O., Delwart E. (2018). Complex virome in feces from Amerindian children in isolated Amazonian villages. Nat. Commun..

