# Supplementary figures and images for: Ten Previously Unassigned Human Cosavirus Genotypes Detected in Feces of Children with Non-Polio Acute Flaccid Paralysis in Nigeria in 2020
Source: Viruses. 2025 Jun 12;17(6):844. doi: 10.3390/v17060844 (PMC12197622; doi:10.3390/v17060844)

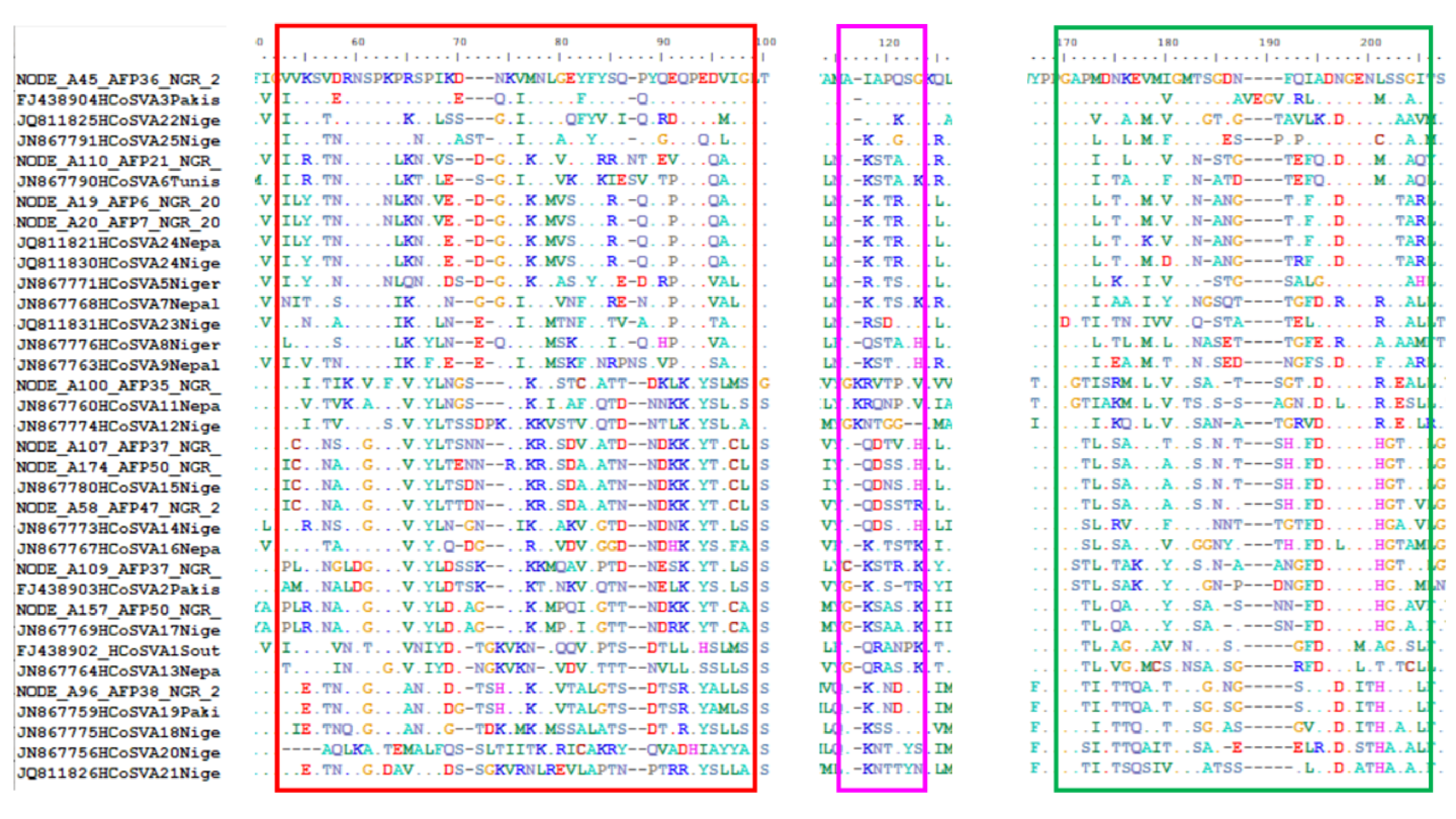

Supplement: Supplementary file 1 [file viruses-17-00844-s001.zip › viruses-3576216-supplementary.tif]
